# Development of an Electrospun Patch Platform Technology for the Delivery of Carvedilol in the Oral Mucosa

**DOI:** 10.3390/nano12030438

**Published:** 2022-01-27

**Authors:** Maria Pardo-Figuerez, Jorge Teno, Alvaro Lafraya, Cristina Prieto, Jose Maria Lagaron

**Affiliations:** 1 Novel Materials and Nanotechnology Group, Institute of Agrochemistry and Food Technology (IATA), Spanish Council for Scientific Research (CSIC), Calle Catedrático Agustín Escardino Benlloch 7, Paterna, 46980 Valencia, Spain; mpardo@iata.csic.es (M.P.-F.); cprieto@iata.csic.es (C.P.); 2 R&D Department, Bioinicia S.L., Calle Algepser 65 nave 3, Paterna, 46980 Valencia, Spain; jteno@bioinicia.com (J.T.); alafraya@bioinicia.com (A.L.)

**Keywords:** carvedilol, electrospinning, nanofibers, mucoadhesive delivery platform, solid amorphous dispersions

## Abstract

The work herein presented aims to develop and characterize carvedilol (CVD) releasable non-water-soluble monolayers and a multilayer patch made of ultrathin micron and submicron fibers for drug delivery into the sublingual mucosa. Firstly, the developed formulations containing CVD within different biopolymers (PDLA, PCL, and PHB) were characterized by scanning electron microscopy (SEM), attenuated total reflectance Fourier transformed infrared spectroscopy (ATR-FTIR), differential scanning calorimetry (DSC), wide-angle X-ray scattering (WAXS), and for their in vitro drug release. SEM micrographs assessed the fiber morphology attained by adding carvedilol. ATR-FTIR spectra revealed good chemical compatibility between CVD and the tested biopolymers, whereas DSC and WAXS confirmed that CVD was in an amorphous state within the biopolymeric fibers. In vitro release studies showed enhanced CVD release kinetics from the electrospun biopolymer monolayers compared to the dissolution rate of the commercial form of the pure drug, except for the slow-releasing PDLA fibers. Finally, the selected CVD-loaded layer, i.e., electrospun PHB, was built into a three-layer patch to tackle mucosa adhesion and unidirectional release, while retaining the enhanced release kinetics. The patch design proposed here further demonstrates the potential of the electro-hydrodynamic processing technology to render unique mucoadhesive controlled delivery platforms for poorly water-soluble drugs.

## 1. Introduction

A large proportion of new drug candidates present poor water solubility, whilst others also present a strong first-pass effect, limiting their use in potential medical therapies [1,2]. In light of this, researchers are focusing their efforts on searching for novel technologies and formulations that can aid to improve the bioavailability and solubility of these active pharmaceutical ingredients (APIs) [3,4,5]. Particularly, interest has been focused on the generation of mucoadhesive delivery platforms to enhance drug permeability and solubility. In this sense, sublingual mucoadhesive systems for API release can become a competitive alternative to traditional delivery systems [6]. Firstly, this route goes directly to the systemic circulation through the internal jugular vein [7], avoiding drug degradation in the gastrointestinal tract or its metabolization via the first-pass effect [6,8]. Additionally, the sublingual mucosa can promote a rapid pharmacological onset, whilst providing a simple and non-invasive approach that facilitates drug administration for patients with swallowing difficulties [9].

The delivery vehicle available for these systems are often manufactured in the form of adhesive gels, particulates [10], tablets [11], and more commonly as patches or films [5,12,13], which are mainly prepared by solvent casting, hot melt, and nanotechnology-based approaches, such as electrospinning. The latter has not only become an attractive technique to generate mucoadhesive systems but also to encapsulate poorly water-soluble ingredients due to its fast evaporation rate, possibility of high drug loading, cost-effectiveness, simplicity of the technique, and versatility using a wide range of drugs, including those which are thermally labile [14]. Besides, a high throughput good manufacturing practice (GMP) approved by pharma authorities for the production of electrospun nanofibers with size control has now been reported, demonstrating the potential of the technology to be up-scaled and used for clinical trials manufacturing [15,16]. 

Carvedilol (CVD) is a non-cardioselective beta-receptor inhibitor, used for cardiovascular diseases and currently presented commercially in the form of tablets for oral administration [17,18]. Unfortunately, the drug presents a poor water solubility and a strong first-pass effect, and so its bioavailability is low (between 25 and 35%) [10]. In an attempt to increase its bioavailability, mucosa administration routes have been proposed, whereby the patch can interact with the mucus layer, finding its way throughout the surface of buccal epithelia. Studies based on hydroxypropyl cellulose-based films were designed with CVD, showing a significant improvement in the bioavailability of carvedilol as a buccal monolayer film when compared to the oral route [5]. Electrospinning was also used to generate electrospun CVD-loaded fibers in water-soluble polymers such as polyethylene oxide (PEO) [2] and polyvinylpyrrolidone (PVP) [19], showing faster dissolution rates when compared to pristine CVD, with almost a complete release in the first 30 min for most of the formulations studied. Although these studies are very meaningful, instant release due to fast polymer dissolution may imply an increase in side effects for certain APIs and fluctuations of the therapeutic concentration in plasma [20]. Similarly, the lack of a backing layer to promote unidirectional release may lead to a percentage of the API released into the saliva rather than adsorbed through the mucosa, thus swallowing that part of the drug during administration.

As an alternative to hydrophilic matrices, non-water-soluble-based polymers have been used in electrospun-based systems to obtain a more controlled release of the API, improving drug use and reducing dose frequency. Synthetic biodegradable polyesters such as polylactide acid (PLA), polyglycolide (PGA), poly-ε-caprolactone (PCL), and the copolymers of lactide (LA), ε-caprolactone (CL), and glycolide (GL) have become popular in the development of drug delivery monolayers due to their biocompatibility and mechanical properties [21,22]. Lately, the use of polyhydroxyalkanoates (PHAs) has also been used for drug release systems due to their biocompatibility and ability to degrade in vivo and in vitro [23,24,25]. Studies from the literature reported the use of electrospun poly-ε-caprolactone fibers with different CVD loadings as potential oral mucosa systems. In this particular study, CVD loadings from 10 to 40% achieved a release of 70 to 90% after 120 h, whilst the slowest drug release was observed in the case of the nanofibers with 50% and 60% of carvedilol, where only 49 to 56% of the drug was released in 120 h. When comparing electrospun fibers with cast films, the CVD release was also much faster from the PCL nanofibers than from the PCL films, highlighting the advantages of using electrospun materials over solvent casting films [21]. The main disadvantage of this system is that it was based on a monolayer of PCL, which would not provide adhesion to the mucosa. To make use of the advantages of using non-water-soluble polymers as reservoir materials whilst maintaining an optimum adhesion to the mucosa, this work presents a three-layer platform approach for CVD, in which an API layer, a mucoadhesive layer, and a backing layer were designed to obtain an efficient drug delivery platform. To this end, the physicochemical characteristics of the solutions such as viscosity, surface tension, and conductivity were investigated. The generated electrospun micron and submicron fibers, also called nanofibers, were then evaluated by scanning electron microscopy (SEM), attenuated total reflectance Fourier transformed infrared spectroscopy (ATR-FTIR), wide-angle X-ray scattering (WAXS), and differential scanning calorimetry (DSC). Finally, the release profile of the drug was studied by UV-Vis spectrophotometry and compared with their film casting counterparts. The release rate of a selected three-layer patch was analyzed by UV-Vis spectrophotometry, and an in vivo study on the adhesion of the three-layer placebo patch was also carried out to confirm a good match between the release obtained and the patch adhesion time.

## 2. Materials and Methods

### 2.1. Materials

Carvedilol (CVD) was obtained from Inke (Castellbisbal, Spain). Poly-ε-caprolactone (PCL, Mw 80,000 Da) was supplied by Perstorp Caprolactones Ltd. (Warrington, UK), Poly(DL-lactide) (PDLA, viscosity midpoint 2.0 dl/g) was supplied by Corbion polymers^®^ (Amsterdam, The Netherlands) and poly(3-hydroxybutyrate) (PHB, under the trade name P226, Mw 426,000 Da) was purchased from by Biomer (Krailling, Germany). For the hydrophilic layer, Polyethylene oxide (PEO, Mw 200,000 Da) was purchased from the Dow Chemical Company (Montomeryville, PA, USA), polyvinylpyrrolidone (PVP, under the trade name Kollidon 90F, Mw 1,250,000 Da) was purchased from BASF (Ludwigshafen, Germany) and ethyl cellulose (EC, viscosity 22 cP, 5% in toluene/ethanol 80:20) was purchased from Sigma-Aldrich (Madrid, Spain). Chloroform (ACS reagent, ≥99.8%) was supplied by Sigma-Aldrich (Madrid, Spain), whilst methanol, ethanol (≥99%), and acetone (≥98%) were supplied by Panreac Química S.L.U. (Castellar del Vallès, Barcelona). The solvent 2,2,2-trifluoroethanol (TFE, ≥99%) was purchased from Alfa Aesar^TM^ (Karlsruhe, Germany) and N,N,-dimethylformamide (DMF, ≥99.8%) was purchased from VWR Chemicals (Leuven, Belgium). All the polymers and reagents were used as received without further purification.

### 2.2. Solution Preparation and Characterization

Solutions were prepared by dissolving each polymer in its corresponding solvent or solvent mixture by continuous stirring at 100 rpm. A concentration of 8 wt.% was used for all cases except for the backing layer, which was 10 wt.%. The list of the developed solutions is gathered in Table 1. Samples were also prepared without CVD for the placebo samples.

The physicochemical properties of the solutions were analyzed in terms of viscosity, surface tension, and conductivity. The conductivity was measured using a Conductimeter Seven2Go S3 with an electrode METLER TOLEDO 742-ISM (Schwerzenbach, Switzerland) and the apparent viscosity was determined by a rotational viscosity meter Visco BasicPlus L from Fungilab S.A. at 50 rpm with an L3 spindle (San Feliu de Llobregat, Spain). Surface tension was measured using a DynoTester tensiometer from Krüss GmbH (Hamburg, Germany). The measurements were carried out in triplicate. Electrospinning is known as a very efficient nanofabrication technique to remove solvents, however, in an industrial application, it has to be analytically proven that remnant solvents remain below the permitted levels. 

### 2.3. Sample Fabrication

#### 2.3.1. Electrospinning and Cast Film Preparation

For the electrospun mats, the electrospinning process was carried out in a high throughput Fluidnatek^TM^ LE-100 equipped with a multi-emitter injector system from Bioinicia S.L. (Valencia, Spain) and an air-conditioned unit. Table 2 shows the electrospinning parameters utilized for each solution. The environmental conditions were kept constant at 25 °C and 30% relative humidity (R.H.) for all the electrospun solutions.

To prepare the cast films loaded with CVD, the solutions were poured at a specific volume into glass Petri dishes and left to dry under controlled humidity and temperature overnight (20 °C and 40% R.H.). 

#### 2.3.2. Three-Layer Patch Fabrication

**Figure 1:**
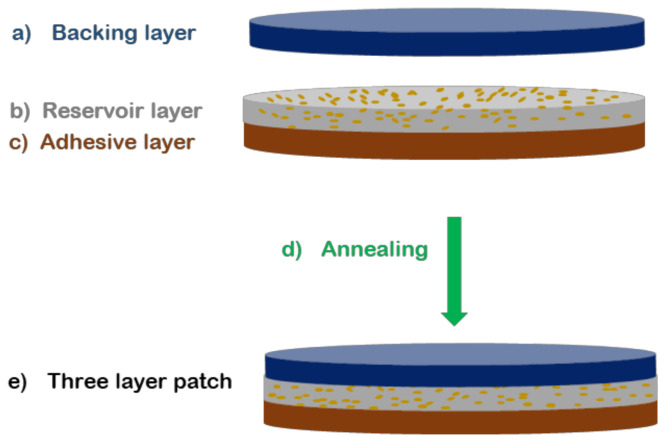
Diagram of multilayer drug delivery approach composed of PCL backing layer (blue), CVD/PHB loaded fibers (middle gray layer, CVD: yellow points), and PEO/PVP/EC adhesive layer (brown).

To prepare the mucoadhesive patch, a three-layer patch structure was prepared using a conceptual design previously developed in our research group [26]. The mucoadhesive layer made of PEO/PVP/EC and PHB with and without CVD electrospun fibers were first electrospun continuously layer by layer. Then, a hydrophobic backing layer made of electrospun PCL fibers was finally deposited onto the previous layers and assembled by applying heat at 50 °C and no pressure for 20 s using a compression molding hydraulic hot press, Carver 4122, Wabash, IN, USA. The multilayer approach can be seen in Figure 1. The generated patch specimens were punched into circles with an area of 2.19 cm^2^.

### 2.4. Characterization

#### 2.4.1. Fiber Morphology

The fiber morphology was analyzed by scanning electron microscopy (SEM) using a Thermoscientific Phenom XL (Bedford, MA, USA) with an electron beam acceleration of 5 kV. The fiber diameter was determined using Phenom ProSuite Software (Bedford, MA, USA) on SEM images. The average fiber diameter was based on at least 100 fibers randomly selected from the software.

To verify the presence and distribution of CVD, microanalysis was performed by energy-dispersive X-ray spectroscopy (EDS, Hitachi High Technologies Corp., Tokyo, Japan).

#### 2.4.2. Attenuated Total Reflection Fourier Transform Infrared Spectroscopy (ATR-FTIR)

The chemical composition of the CVD-loaded electrospun fibers was evaluated by attenuated total reflection Fourier transformed infrared spectroscopy (ATR-FTIR). Fourier transform infrared (FTIR) spectra were collected coupling the attenuated total reflection (ATR) accessory Golden Gate of Specac, Ltd. (Orpington, UK) to the Tensor 37 FTIR equipment (Bruker, Germany). Single spectra were collected in the wavelength range from 4000 to 600 cm^−1^ by averaging 10 scans at a resolution of 4 cm^−1^.

#### 2.4.3. Differential Scanning Calorimetry (DSC)

The physical state of CVD-loaded electrospun fibers was studied by differential scanning calorimetry (DSC) on a DSC-8000 analyzer from PerkinElmer, Inc. (Waltham, MA, USA), equipped with a cooling accessory Intracooler 2, also from PerkinElmer, Inc. Approximately 3 mg of each sample were placed in standard aluminum pans and heated from 30 to 220 °C and at a rate of 10 °C/min using a nitrogen flow of 20 mL/min as the sweeping gas. The potential curvature of the endotherms was corrected with these of an empty pan.

#### 2.4.4. Wide-Angle X-ray Scattering (WAXS)

Wide-angle X-ray scattering measurements were performed using a Bruker AXS D4 Endeavor diffractometer. The samples were scanned at room temperature in reflection mode using incident Cu K-alpha radiation (Cu Kα = 1.54 Å), while the generator was set up at 40 kV and 40 mA. The data were collected over a range of scattering angles (2Θ) in the 5–40° range.

#### 2.4.5. In Vitro Drug Release

Drug release was evaluated using an ultraviolet/visible spectrophotometer DINKO UV4000 (Barcelona, Spain). For this, membranes containing 157.23 ± 0.01 µg/cm^2^ of CVD were used. The release of CVD-loaded electrospun fibers into the medium was monitored by measuring the absorbance at 240 nm at predetermined times. Each electrospun sample was weighed and adjusted with the corresponding volume of aqueous phosphate buffer solution (pH = 6.8) whilst stirring at 100 rpm at 37 °C, to achieve an initial CVD concentration of 8.5 ± 0.5 µg/mL. The electrospun samples were attached to supports and lowered into the solution media. In the case of the three-layer approach, the patch was placed with the adhesive layer facing towards the buffer solution to somehow mimic the initial contact with the mucosa. For the standard curves, a CVD stock solution was prepared according to the literature and diluted with an aqueous phosphate buffer solution (pH 6.8) [27]. The release experiments and the calibration curves were carried out in triplicate (y= 0.1191x + 0.0151, R^2^ = 0.9998).

##### Determination of CVD Loading in the Fibers

To determine the experimental loading of CVD within the fibers, approx. 0.5 mg of loaded electrospun fibers were dissolved in 5 mL of TFE to fully dissolve both carvedilol and its corresponding polymer. The resulting solution was stirred at 100 rpm for an hour and then analyzed using an ultraviolet/visible spectrophotometer (DINKO UV4000 (Barcelona, Spain). The reading was compared against a calibration curve produced using standard samples of 1–20 µg/mL of CVD in TFE. The absorbances at 240 nm were recorded and the experimental *CVD Loading* (%) in the fibers was calculated as follows:

(1) $$CVD\;Loading\;\left(\%\right)=\frac{md}{mp}\times 100$$

where *m_d_* is the mass of the drug obtained experimentally as described above, and *m_p_* is the total mass of the sample analyzed.

#### 2.4.6. Patch Residence Time Study

The adhesive capability of a placebo PHB three-layer patch (without CVD) was investigated in vivo. For this, the placebo patch specimens were tested by 10 healthy human volunteers (5 males and 5 females) with ages between 25 and 55 years old. Similar to other studies [28,29], the volunteers were asked to apply the multilayer patches of 2.19 cm^2^ in the sublingual mucosa region for 5 s with applied pressure. To assess the overall comfortability of the users, the volunteers were asked to fill in a five points’ hedonic scale, indicating: 1—very uncomfortable, 2—uncomfortable, 3—neutral, 4—comfortable, and 5—very comfortable. Additionally, the residence time of the patch was defined as the time interval between the application of the patch and the time point where it moved, detached, or could not be felt any more at the site of application. During the test period, volunteers were allowed to drink, eat and speak.

## 3. Results and Discussion

### 3.1. Physicochemical Solution Properties

It is well known that the physicochemical properties of polymer solutions play an important role in electrospinning processability and therefore in the final fiber morphology of the material [30,31]. Additives such as surfactants, other polymers, and active ingredients can also affect the properties of the solution, modifying the processability of electrospinning. Thus, the viscosity, surface tension, and conductivity of the solutions with and without CVD were measured and summarized in Table 3. Both viscosity and conductivity values increased with the addition of CVD, which could be due, on one hand, to the presence of more solid content in the solution and, on the other hand, to the presence of the ionic form of the drug in the solution. Surface tension decreased with the presence of CVD for all the solutions, which could be due to weak interactions between CVD, solvents, and the polymers. Analyzing the solution properties can anticipate processability issues during electrospinning; however, it is difficult to assess the effect of a single property without considering the impact of the others [32,33]. In this case, these observations were not translated into processability issues and in fact, solutions with and without CVD were electrospun with the same process parameters, as stated in Table 2. 

### 3.2. Fiber Morphology

**Figure 2:**
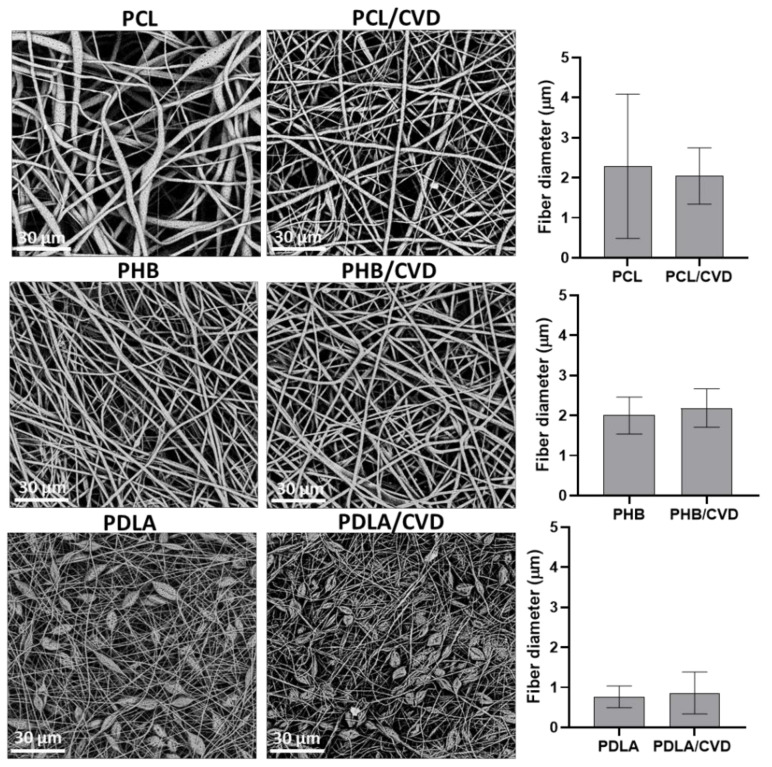
(**Left**) Scanning electron microscopy (SEM) micrographs of the different PCL, PHB, and PDLA fibers with and without CVD. (**Right**) The average fiber diameter of the different loaded PCL, PHB, and PDLA fibers with and without CVD. Values are presented as the mean ± S.D.

The CVD-loaded electrospun fibers prepared with different polyesters were analyzed by SEM (see Figure 2). The pristine PCL and PCL/CVD fibers presented a visible difference in morphology, with the pristine PCL fibers presenting large porous beads across the fibrous material. The addition of CVD into electrospun PCL fibers resulted in uniform and smooth fibers, which may indicate that the presence of CVD promoted the elimination of beads, most likely due to a slight increase in viscosity and conductivity. This was also translated into a slight reduction in fiber diameter, which could be attributed to a more homogeneous dispersion across fiber size with the presence of CVD. Beaded fibers are certainly bigger in diameter than uniform fibrous structures and also contain a higher dispersity in fiber size. The PHB fibers and PHB/CVD fibers presented uniform smooth morphologies in both cases, whilst PDLA fibers and PDLA/CVD mats presented a beaded morphology, most likely due to the low molecular weight of the polymer [34] and hence the low viscosity of the solution (Table 3). In terms of fiber diameter, the presence of CVD slightly increased fiber diameter for both PHB/CVD and PCL/CVD, although without major differences. The ultrathin fibers obtained for PCL and PHB had an average micron size, but a fraction of those fibers were observed to fall below the micron range into the nanoscale. For PDLA, the average fiber size was already below the micron size. For all the samples, SEM micrographs did not present any visible particles at the surface of the fibers, which could be a qualitative indication of the effective entrapment of CVD into the fibers, in good agreement with previous studies [19,21].

### 3.3. ATR-FTIR

**Figure 3:**
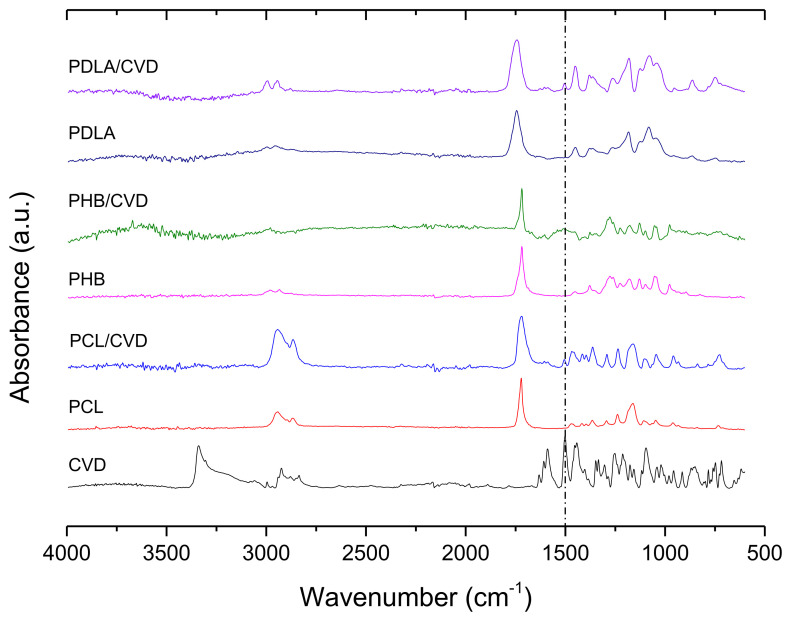
ATR-FTIR spectra of samples with different polymer matrices (PCL; PCL/CVD, PHB, PHB/CVD, PDLA; PDLA/CVD) and commercial CVD. For comparison, the C=C stretching bond in the aromatic ring of CVD has been highlighted with a dashed-dotted line.

Figure 3 shows ATR-FTIR spectra which were used to analyze possible interactions between CVD and the polymers, and also to confirm the drug’s presence in the fibers. For commercial pure CVD, the characteristic vibration modes can be found at 3340 cm^−1^, corresponding to the O-H and N-H stretching vibration peaks merged together. The peaks at 2923 and 2835 cm^−1^ can be assigned to the C-H stretching and the peaks at 1502 and 1589 cm^−1^ were assigned to the C=C stretching bond in the aromatic ring (dashed line pointed in Figure 3). The vibration of C–N is observed at 1454 cm^−1^, whilst the vibration associated with the phenyl group ring C–C is in the range of 1403–1256 cm^−1^ [18,35,36].

The main bands observed in the PCL and PCL/CVD spectra were attributed to CH_2_ vibrations at 2946, 2870, and 2821 cm^−1^ whilst C=O and C–O–C stretching can be seen at 1720 cm^−1^, at 1238 cm^−1^, and 1161 cm^−1^, respectively [37]. PHB and PHB/CVD samples showed their characteristic bands at 1718 cm^−1^, which is related to C=O stretching vibrations. The CH_3_ asymmetric and symmetric deformation was observed at 1452 cm^−1^ and 1379 cm^−1^ respectively, whilst C–O–C stretching was observed at 1278 cm^−1^, 1224 cm^−1^, and 1178 cm^−1^, as also reported in other studies [38].

PDLA and PDLA/CVD spectra showed the characteristic bands at 2999 and 2956 cm^−1^ attributed to C-H stretching. The band at 1743 cm^−1^ was associated with the stretching of the ester carbonyl group (C=O), whilst CH_3_ asymmetrical stretching appeared at 1450 cm^−1^. The C–O–C stretching was identified at 1080 cm^−1^, in agreement with other reports [39] (Figure 3).

From this figure, it can be discerned that CVD-loaded biopolyester fibers (PCL/CVD, PHB/CVD, and PDLA/CVD) showed the characteristic absorbance bands associated with the vibration of aromatic stretching C=C of CVD (dashed line pointed in Figure 3), confirming the presence of CVD in the different polymer matrices. However, other characteristic vibration modes of CVD seem to overlap with the vibration modes of the polymer matrices. Additionally, the CVD characteristic peak at 3340 cm^−1^ attributed to the N–H stretching is not observed in any of the CVD-loaded fibers. This could be related to the potential interaction between the –NH group of CVD and the –CO groups of the matrices as previously reported [40].

### 3.4. Thermal Analysis (DSC)

**Figure 4:**
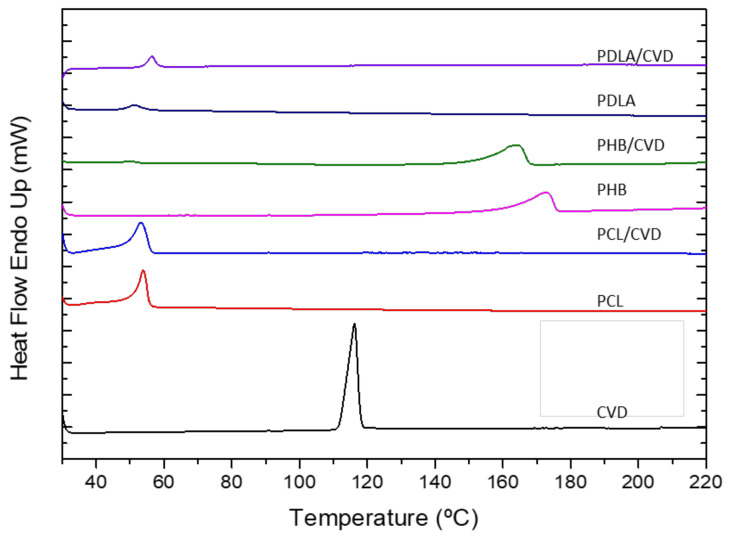
DSC thermograms of different polymer matrices with and without CVD (PCL; PCL/CVD; PHB, PHB/CVD; PDLA; PDLA/CVD) and the commercial CVD powder.

Differential scanning calorimetry (DSC) was used to analyze the physical state of the drug and polymer in the electrospun fibers. Figure 4 shows the DSC data of the different electrospun polyesters with and without CVD as well as the thermal run for the commercial CVD. The thermogram of CVD showed an endothermic peak around 116.25 °C with a ΔH_m_ of 98.10 J/g, revealing its crystalline nature [2]. In all CVD fiber-loaded formulations, the melting point of the API was not detected, suggesting that CVD is in an amorphous form inside the fibers. Additionally, differences in the polymer melting point were observed for the electrospun biopolymers in the presence of CVD. In the case of the pristine PCL and PCL/CVD fibers, there was a melting temperature (T_m_) of 53.90 °C and 53.17 °C, respectively, suggesting a slight shift to a lower T_m_ for the CVD-loaded fibers. Similar behavior can be observed for the PHB fibers and PHB/CVD electrospun samples, where the melting temperature shifted towards a lower temperature for the PHB/CVD fibers. Thus, the T_m_ value of the neat PHB was 172.48 °C, whereas the value of the PHB/CVD fibers was 164.21 °C. Such a shift and broadening in the melting peak of these polymers indicate that the presence of CVD molecules induced a plasticizing effect, which interferes with the biopolymer crystallization process [41].

In the case of the PDLA samples, the glass transition temperature (T_g_) of the CVD-loaded electrospun fibers shifted slightly towards higher temperature, again suggesting that the API molecules provoked some plasticization effect on the polymer. Thus, the presence of the API in the electrospun polymer fibers seems to allow the polymer chains to move more freely, leading to a somewhat higher T_g_ [42].

For all of them, the absence of the CVD melting peak for all the tested mixtures can be clearly observed. Similar work presented by Potrč et al. [21] showed the absence of CVD peaks for PCL-CVD fibers with loadings from 10 to 60%, suggesting that the presence or absence of this peak was not ascribed to a low loading of CVD in the fibers. However, it is noteworthy to mention that low melting point polymers like PCL could melt first in the DSC and dissolve the CVD crystals that may be at the surface of the nanofibers, thus leading to the absence of previously CVD in its crystalline form [21]. The study presented here did not show any visible residual CVD on the SEM images (see Figure 2). However, WAXS analyses were performed to confirm these results.

### 3.5. Wide-Angle X-ray Scattering (WAXS)

**Figure 5:**
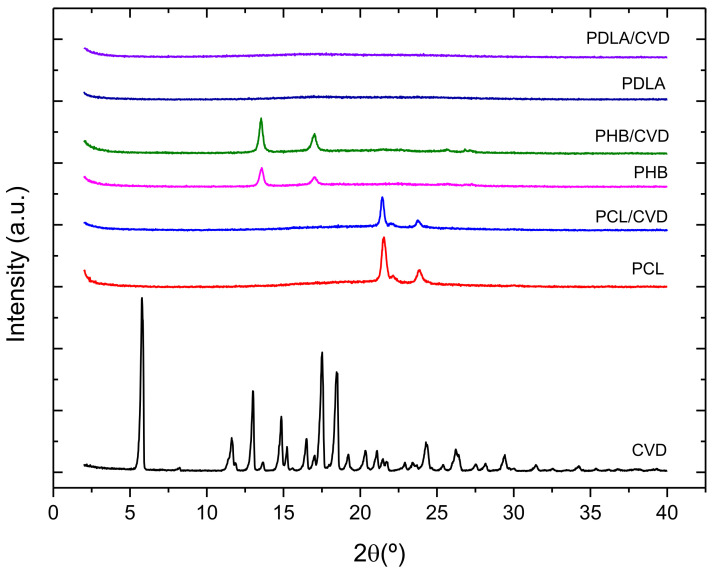
WAXS spectra of the different polymer matrices with and without CVD (PCL; PCL/CVD; PHB, PHB/CVD; PDLA; PDLA/CVD) and the commercial CVD powder.

The WAXS patterns of the CVD commercial powder and loaded CVD fibers were also taken to further assess the crystalline phase of the materials. Figure 5 shows the diffractograms of the CVD-loaded fibers and commercial CVD powder. The CVD diffractogram showed several sharp peaks, attributed to the crystalline nature of the drug, as also observed in other studies [18]. As per the CVD-loaded electrospun mats, the intensity peaks of PCL/CVD and PHB/CVD were associated with the crystallinity of the polyesters. On the other hand, PDLA/CVD fibers did not show any peaks, highlighting the amorphous state of this polymer. Interestingly, the presence of the CVD diffraction peaks was not observed in any of the diffractograms of the CVD-loaded fibers. Furthermore, the lack of CVD peaks remained for all the tested loaded fibers after 2 months of storage at 30% R.H. (results not shown). The combination of the DSC and WAXS data clearly indicated that CVD was in the form of a solid amorphous dispersion within the electrospun materials. 

### 3.6. In Vitro CVD Release Rate from Monolayers

The theoretical CVD loading was set to 10 wt% in this study obtaining a ratio of 90:10 polymer:API in the fiber. To quantify the attained CVD loading present in the fibers, the samples were fully dissolved and measured by UV-Vis spectroscopy. The results indicated that the CVD loading in the fibers was 9.41 ± 0.32% for PCL-CVD-loaded fibers, 10.45 ± 0.39% for PHB fibers, and 10.11 ± 0.54% for PDLA fibers. This indicates a good agreement with the expected values, suggesting a close to 100% process efficiency. 

**Figure 6:**
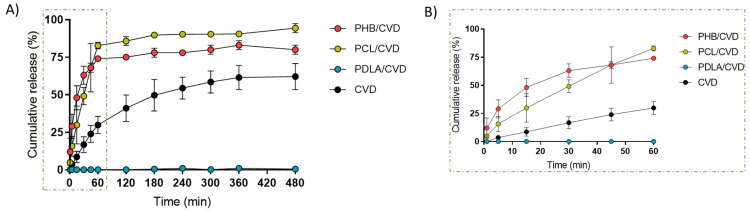
(**A**) CVD release profiles from fibers PCL/CVD, PHB/CVD, PDLA/CVD, and from commercial CVD. (**B**) CVD release within the first hour. Graphs are presented as the mean ± S.D. (*n* = 3).

Figure 6 gathers the release profile of the PCL/CVD, PHB/CVD, and PDLA/CVD fibers vs. CVD commercial powder. The fibrous PHB/CVD and PCL/CVD samples presented a two-stage release pattern with approximately 70–80% release of CVD (approximately 258 µg) content after an hour, followed by a steady slow-release phase. Such initial burst release may be attributed to the diffusion out of CVD near the fiber surface. For both types of electrospun fibers, a faster CVD release can be seen when compared with the release kinetics of CVD pure powder, where only 25% is measured after the first hour. 

Interestingly, a previous study based on PCL electrospun fibers containing 10% of CVD released only 30–40% after an hour. In our study, up to 75% was released after 1 h. This difference can be attributed to the different fiber characteristics such as diameter and morphology, pH medium, drug dispersion across the fibers, etc. [21].

As for the PDLA/CVD electrospun fibers, a much slower release rate was observed, with barely a 2% cumulative release after 8 h. The amorphous state of the API in this matrix was observed by DSC and WAXS (Figure 4 and Figure 5), and so such a slow release could be attributed to the glassy state of PDLA at the temperature of the experiment [43,44,45]. Drug molecules efficiently distributed throughout a polymer in a glassy-like state will only be released when the polymer degrades, thus resulting in a very slow release [46].

**Figure 7:**
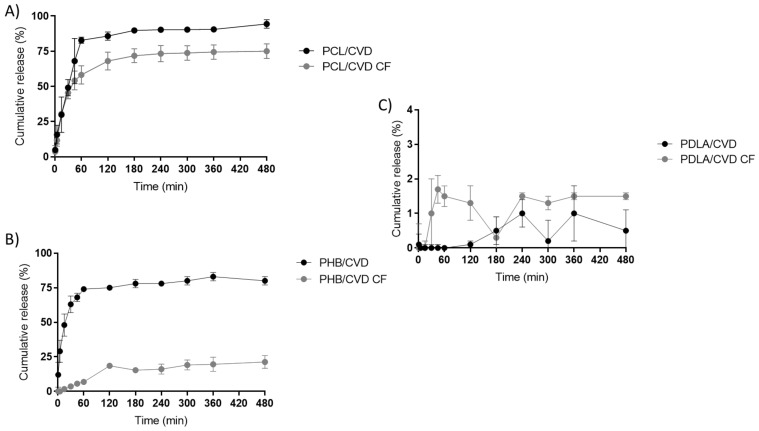
(**A**) Release rate profiles of PCL/CVD electrospun fibers and PCL/CVD CF cast films (indicated as CF). (**B**) Release rate profiles of PHB/CVD electrospun fibers and PHB/CVD CF cast films. (**C**) Release rate profiles of PDLA/CVD electrospun fibers and PDLA/CVD CF cast films. Graphs are presented as the mean ± S.D. (*n* = 3).

In order to assess the comparative effectiveness of the electrospinning technology with other forms of manufacturing films, similar films produced by the solvent casting method were also investigated for the delivery of CVD. Figure 7 shows the drug release profile of PCL/CVD, PDLA/CVD, and PHB/CVD alongside their casting film counterparts (PCL/CVD CF, PDLA/CVD CF, and PHB/CVD CF, respectively). When compared with their homologous cast films, the electrospun fibers released CVD faster, but for PDLA fibers. The total release after 8 h was typically higher for the electrospun PHB and PCL fibers than for the cast films. Likewise, a two-phase release was observed, with an initial burst followed by a slower release. Overall, it can be stated that electrospun fibers, especially PHB/CVD, had a faster CVD release behavior than their cast film counterparts, except for the case of PDLA, which presented a similar behavior of slow-release rate for both fibers and cast films. Polymer characteristics such as crystallinity, T_g_, material density, CVD distribution, and phase morphology will certainly affect the release kinetics for the same chemistry [47].

### 3.7. Characterization of the PHB/CVD Three-Layer Patch

Mucoadhesive drug delivery platforms have proven very efficient for increasing drug bioavailability whilst improving patient compliance [4,48,49]. However, these patches or films can be often comprised of non-water-soluble monolayer polymers, which make the mucoadhesion of the patch difficult. Likewise, unidirectional drug release towards the oral mucosa is necessary to avoid back release into the mouth. To this end, a multilayer patch was prepared with the PHB/CVD-loaded nanofibers as the release model matrix, since initially a slightly faster release was appreciated (Figure 6B) in comparison with the other matrices. Additionally, the use of PHB has not been extensively explored as a drug delivery matrix, and so more studies down this route could open new avenues of applied research in the pharma field. As described in Figure 1, the patch consisted of a middle layer of PHB/CVD as the API reservoir layer, a backing layer of annealed impermeable electrospun PCL fibers to avoid back diffusion, and a layer blend of PEO/PVP/EC to provide mucoadhesion.

**Figure 8:**
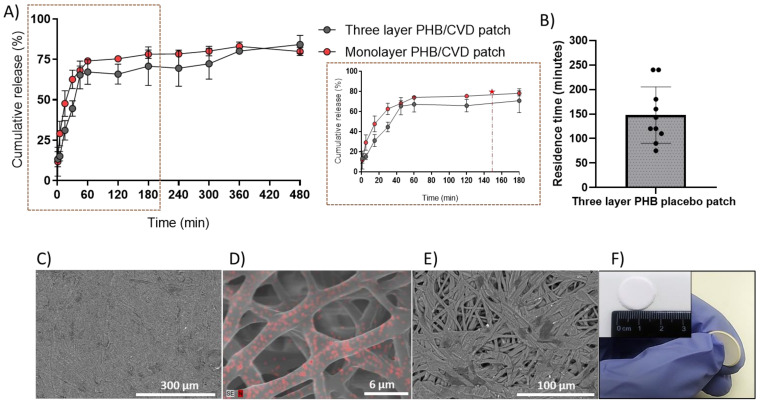
(**A**) Release rate profile of PHB/CVD as a mono and three-layer patch. The release rate profile has been zoomed out to analyze the differences across the profiles in the first two hours, as well as to correlate the release kinetics of the API with the residence time of the patch in the mucosa (red star and dashed line). (**B**) In vivo sublingual mucoadhesive performance of the PHB multilayer patch without CVD (PHB multilayer patch). (**C**) Scanning electron microscope (SEM) micrographs of the backing layer (PCL), (**D**) SEM micrographs of PHB-CVD layer processed by energy-dispersive X-ray spectroscopy (EDS). (**E**) SEM micrographs of the mucoadhesive layer (60PEO/30PVP/10EC) and (**F**) macroscopic image of the multilayer patch system. Graphs are presented as the mean ± S.D. (*n* = 3).

Figure 8A highlights the cumulative drug release of PHB/CVD multilayer patch against the PHB/CVD monolayer. As shown in the zoomed figure, there is a somewhat slower release rate for the multilayer approach during the first 30–60 min, most likely due to the cross-diffusion of the buffer media and CVD across the mucoadhesive layer. Thereafter, a similar release behavior was observed across the mono and the multilayer approach, indicating that the release profile is reproducible and effective, even with the presence of extra layers. 

For the in vivo study, the same patch as mentioned above was prepared without CVD. The PHB placebo patch was placed in the sublingual mucosa of a number of volunteers and these were assessed in terms of comfortability and residence time (Figure 8B). Overall, most volunteers reported a minor uneasiness at the time of applying the patch, but a few minutes later all volunteers felt comfortable while wearing the patch, with an average of 4 ± 1 on the hedonic scale. On the other hand, the patch adhesion time was approximately 2.47 h (147.8 ± 57.6 min), albeit a large distribution across the volunteers was found, probably because no restrictions were given to the volunteers in terms of eating and drinking during the study. As per the correlation between the adhesion and drug release, it can be concluded that the adhesive layer allows an average sufficient adhesion for a release of approximately 75–80% of the CVD, which would be equivalent to 118–126 µg/cm^2^ of CVD (see red star and dashed line in Figure 8A). If needed, larger doses could be applied by increasing patch area or thickness. Lastly, the morphology of the different layers of the multilayer patch is shown in Figure 8C–E. The PCL backing layer is presented as a rather continuous film with a highly reduced porosity due to the fibers coalescence process that occurs during annealing (Figure 8C), whilst the mucoadhesive layers present a microfibrous structure that will enhance the adhesion properties to the mucosa (Figure 8E). The PHB-CVD layer was analyzed by EDS (Figure 8D), and the distribution of CVD was correlated to the presence of nitrogen (N, red spots) within the electrospun fibers since it is a unique characteristic element of CVD. From the image, highly dispersed and distributed submicron CVD can be inferred across the electrospun PHB fibers. An assembled patch presenting the whole multilayer structure is then highlighted in Figure 8F.

## 4. Conclusions

The work herein presented demonstrates the feasibility of using the nanofiber fabrication electrospinning technique as a solubility enhancer technology for poorly water-soluble drugs. Various biopolymers, such as PCL, PHB, and PDLA were fabricated to generate electrospun active monolayers to deliver directly to the oral mucosa poorly water-soluble drugs like CVD. The amorphous physical state of the encapsulated drug was confirmed by DSC and WAXS, whilst the ATR-FTIR spectrum confirmed that CVD was compatible with the different biopolymers used. Results of the drug release kinetics from the electrospun biopolymer monolayers suggested an enhancement in the release rate of CVD compared to the actual dissolution kinetics of the commercial pure CVD, except for the slow-releasing PDLA. Electrospun monolayers of PCL and PHB were also seen to release at a faster pace than their solvent cast counterparts. From these results, a multilayer patch approach with CVD-loaded PHB was selected and designed. This oral mucosa patch delivery route was selected since CVD undergoes a heavy first-pass effect, and so buccal delivery systems could overcome this issue. In this case, the use of an adhesive layer resulted in a somewhat slower release at short times compared to the monolayer, suggesting that the drug release can be further modulated not only from the reservoir layer but also by patch design. When carrying out studies with the three-layer placebo patch, the mucoadhesion data suggested an overall adhesion of the patch between 2 and 3 h. Nevertheless, the advantages of these kinds of platforms are that shape and size can be carefully designed and thus different mucoadhesion and dose requirements can be easily adapted.

The obtained results indicate that electrospinning is further confirmed as a very promising technology to generate drug delivery platforms made of ultrathin fibers for poorly water-soluble drugs. Furthermore, the developed three-layer patch strategy shows good potential as a sublingual drug delivery system. For this particular case, the sublingual strategy may be more appropriate since the drug can enter the systemic circulation directly.

## Figures and Tables

**Table 1:** Composition of the different polymer solutions. The same solutions were used to prepare the cast films.

Sample	Polymer Matrix	Polymer/CVD Ratio (*w*/*w*)	Solvents Ratio (*w*/*w*)
PCL	PCL	-	Chloroform/Methanol (90/10)
PCL/CVD		90/10	
PHB	PHB	-	TFE
PHB/CVD		90/10	
PDLA	PDLA	-	Acetone/DMF (80/20)
PDLA/CVD		90/10	
Backing layer	PCL	-	Chloroform/Methanol (90/10)
Mucoadhesive layer	PEO/PVP/EC	60/30/10	Distilled H_2_O/Ethanol (50/50)

**Table 2:** Electrospinning parameters of polymeric solutions with and without CVD. V+ indicates voltage on the injector and V- indicates voltage on the collector.

Sample	Polymer Matrix	Flow Rate (mL/h)	Voltage V+/V− (kV)	Needle-to Collector Distance (cm)
PCL	PCL	20	20/−20	30
PCL/CVD				
PHB	PHB	20	25/−5	25
PHB/CVD				
PDLA	PDLA	15	20/−20	30
PDLA/CVD				
Backing layer	PCL	20	15/−2	15
Mucoadhesive layer	PEO/PVP/EC	26	30/−20	28

**Table 3:** Characterization of the prepared CVD and polymeric solutions in terms of conductivity (µS/cm), surface tension (mN/m), and viscosity (Cp).

Sample ID	Polymer Matrix	Polymer/API Ratio (*w*/*w*)	Conductivity (µS/cm)	Viscosity (Cp)	Surface Tension (mN/m)
**PCL**	PCL	-	0.14 ± 0.01	328.5 ± 8.3	46.7 ± 4.9
**PCL/CVD**	PCL	90/10	0.59 ± 0.10	346.3 ± 7.4	30.1 ± 2.0
**PHB**	PHB	-	10.80 ± 0.20	369.4 ± 16.7	47.2 ± 1.9
**PHB/CVD**	PHB	90/10	33.68 ± 0.80	410.4 ± 6.3	25.7 ± 0.6
**PDLA**	PDLA	-	0.36 ± 0.01	46.3 ± 3.3	31.2 ± 1.3
**PDLA/CVD**	PDLA	90/10	0.41 ± 0.02	66.6 ± 4.1	24.0 ± 0.5

## Data Availability

The data presented in this work are available upon request from the corresponding author.
